# An Interval-valued intuitionistic Fuzzy group decision-making approach based on projection measurement extended VIKOR for patent quality evaluation

**DOI:** 10.1371/journal.pone.0323019

**Published:** 2025-05-27

**Authors:** Xiaojun Xie, Saratha Sathasivam, Hong Ma

**Affiliations:** 1 School of General Education, Guangzhou College of Technology and Business, Guangzhou, China; 2 School of Mathematical Sciences, Universiti Sains Malaysia (USM), Penang, Malaysia; 3 School of Financial Mathematics and Statistics, Guangdong University of Finance, Guangzhou, China; Anhui University, CHINA

## Abstract

Patent quality assessment is a multi-attribute decision-making problem. The increasingly complex social environment makes group decision-making based on interval-valued intuitionistic fuzzy (IVIF) information more and more important. In group decision-making, considering using a comprehensive projection measure to calculate the similarity between two decision matrices is one of the important steps. In order to make up for the deficiencies of the classical projection measure and the existing normalized projection measure in measuring the similarity between two IVIF vectors and optimize the group decision-making performance of the VIKOR method, this study proposes: (1) a new definition of subtraction of IVIF numbers, which further enriches the theoretical basis of IVIF numbers; (2) a new normalized projection measure to measure the similarity between two IVIF matrices; (3) combined with the regret matrix to store group regret information, which enhances the comprehensive ability of the classical VIKOR technique, and then establishes a comprehensive GDM method for patent quality assessment. Finally, a specific patent quality assessment example in the existing literature is used for data experiment. Through static and dynamic experimental comparative analysis, the feasibility and effectiveness of the proposed method are illustrated, and it can almost produce a stable ranking. The research in this paper not only provides an effective group decision-making tool for patent quality assessment, but also provides theoretical support and practical guidance for fuzzy group decision-making problems in other fields.

## 1. Introduction

### 1.1 Background

In today’s era of globalization and rapid technological change, patents, as an important symbol of enterprise innovation, have become the core assets that drive enterprise competitiveness, technological progress, and market expansion [1]. The quality of patents directly affects an enterprise’s technological innovation ability, market benefits, and intellectual property protection level, and thus has a profound impact on the long-term development of the enterprise. Therefore, patent quality assessment has become an important issue that needs to be urgently addressed in academia and the business community. However, with the surge in the number of patent applications, a large number of patents with varying qualities have emerged in the market, including junk patents and problem patents. These low-quality patents not only waste enterprise resources but may also have a negative impact on enterprise technological innovation and competitiveness [2]. Therefore, how to effectively and scientifically evaluate the quality of enterprise patents has become a key topic in patent management and intellectual property strategy.

At present, research on the assessment of enterprise patent quality mainly focuses on aspects such as technological level, market benefits, and legal protection [3]. Most traditional enterprise patent quality evaluation methods rely on quantitative indicators, such as the patent grant rate, citation frequency, and patent duration. Although these indicators can reflect the technological advancement and market potential of patents to a certain extent, they often overlook the complexity, uncertainty, and multi-dimensional characteristics of patent assessment [4]. Therefore, constructing an enterprise patent quality assessment method that comprehensively considers multiple quantitative and qualitative indicators and addressing the subjectivity, fuzziness, and uncertainty in the assessment process is a difficult problem that urgently needs to be solved in current research and practice.

### 1.2 Motivation of the group decision-making method based on VIKOR

As mentioned above, in theory, patent quality assessment is a multi-attribute decision-making (MADM) problem. In the real world, patent quality assessment often also involves the opinions of multiple stakeholders (such as experts, enterprise managers, technicians, etc.) [5]. In this sense, patent quality assessment is a group decision-making (GDM) problem [6]. GDM can effectively integrate the opinions of different decision-makers, avoid the bias of a single decision-maker, and provide more objective and comprehensive assessment results. This study adopts a systematic GDM method for patent quality assessment. Among them, the ViseKriterijumska Optimizacija I Kompromisno Resenje (VIKOR) method is a classical decision-making method [7], which is especially suitable for GDM and MADM problems. It can find a compromise solution by optimizing multiple evaluation criteria. In the context of patent quality assessment, the introduction of the VIKOR method can effectively deal with the multi-dimensional and complex problems in patent quality assessment. Therefore, the VIKOR-based GDM method has become an important tool for solving the problem of enterprise patent quality assessment. However, this study identifies a research gap: in current enterprise management, there is a lack of a VIKOR-based GDM method for evaluating corporate patent quality. This gap has led to the following Research Question:

#### Research question 1.

How can a scientifically sound and reasonable VIKOR-based GDM method be constructed within the MCDM framework to assess corporate patent quality?

This research gap motivates the objective of this study. The present work aims to develop a new VIKOR-based GDM method for evaluating corporate patent quality. Through a systematic review and analysis of existing VIKOR-based GDM methods, this study also identifies that while current VIKOR techniques incorporate regret measurement, they lack a specific regret matrix to represent and store regret information (similar to a utility matrix). This leads to the following Research Question:

#### Research question 2.

How can a specific regret matrix be computed to represent and store regret information? Addressing this question is highly meaningful for further enhancing the overall capability of the VIKOR technique.

### 1.3 Motivation of the GDM method based on IVIF

Traditional GDM methods mostly rely on deterministic data [8]. However, in the process of patent quality evaluation, due to the complexity and uncertainty among various evaluation dimensions, traditional GDM methods may not fully meet the complex assessment needs. Therefore, considering the GDM method based on interval-valued intuitionistic fuzzy (IVIF) numbers, it can better adapt to the uncertainty problem in enterprise patent quality assessment [6]. IVIF numbers simultaneously contain information on membership degree, non-membership degree, and hesitancy degree, and all are in the form of interval values, which makes it more flexible and practical in dealing with fuzziness and uncertainty[9]. In a sense, it can be said that IVIF number is more in line with human thinking in describing uncertain information. In this study, the following research question exists:

#### Research question 3.

How to utilize IVIF information to evaluate uncertain indicators such as the technical level and market benefits of enterprise patent quality.

### 1.4 Motivation of the VIKOR method based on projection measures

The classical VIKOR method [10] usually uses the Euclidean distance or Hamming distance to measure the closeness between the evaluated object and the ideal reference solution. However, distance measurement has certain limitations in decision-making, which has led to the application of projection measurement. First, distance measurement is highly sensitive to data distribution and is easily influenced by groups with high variance, leading to misjudgments of data structure. For example, in investment decision-making, when different projects have significantly different distributions of risk and return, distance measurement may excessively emphasize the influence of high-risk projects while overlooking the potential value of other projects. Second, distance measurement primarily focuses on the absolute distance between data points, often neglecting the directionality of data and the interrelationships between variables. However, in applications such as market trend analysis, the direction of data changes and interactions between variables are crucial to decision-making, and relying solely on distance measurement may result in inaccurate trend recognition. Finally, in high-dimensional decision spaces, distance measurement faces the “curse of dimensionality” problem. As the dimensionality increases, the distances between data points become indistinguishable, reducing its effectiveness. For example, in multi-attribute product evaluation, high-dimensional space increases the complexity of distance-based evaluation methods, making it difficult to accurately measure differences between products, thereby affecting decision accuracy. Therefore, projection measurement provides a more comprehensive and effective alternative that better adapts to complex decision-making environments while reflecting both the magnitude and direction between two decision matrices [11]. Consequently, the VIKOR method extended based on projection measurement has gained increasing attention from researchers [12,13]. However, this study finds that both the classical projection measurement and the standardized projection measurement in existing literature are not always reasonable under the interval intuitionistic fuzzy setting (see Examples 1 and 2 in Section 3), which raises the following Research Question:

#### Research question 4.

How can new projection measurement formulas be further developed to address the shortcomings of classical projection measurement and the standardized projection measurement formulas in existing literature? Improving these formulas is crucial for enhancing the applicability and accuracy of projection measurement.

### 1.5 Research motivation

To address the aforementioned Research Questions, this study has the following research motivations:

(1) To fill the gap in **Research Question 1**, this study will propose a new VIKOR-based GDM method for assessing the quality of enterprise patents.
(2) To solve **Research Question 2**, this study will define the subtraction operation of IVIF numbers and subsequently construct a specific regret matrix to store regret information.
(3) To solve **Research Question 3**, this work intends to employ IVIF numbers to represent the evaluation information provided by decision-making experts. That is, to facilitate the expression of individual opinions, decision-makers can use IVIF information to articulate their judgments.
(4) To solve **Research Question 4**, this study seeks to develop a novel normalized projection measurement formula to assess the proximity between two evaluation matrices of IVIF numbers.

### 1.6 Organization

The organization of the rest of this study is as follows: Related research work is introduced in Section 2. Relevant preliminary knowledge is presented in Section 3. An enterprise patent quality assessment method is proposed in Section 4. The procedures and basic steps of the enterprise patent quality assessment method in this paper are shown in Section 5. An application example and experimental analysis of enterprise patent quality assessment are given in Section 6. The research conclusions and future research directions of this paper are finally summarized in Section 7.

## 2. Related work

This section provides a review of related research in two aspects: the first focuses on fuzzy group decision-making methods based on the VIKOR approach, and the second examines decision-making methods based on projection measures. Finally, the main shortcomings and limitations of these methods are summarized.

The VIKOR method was proposed by Opricovic in 1998 [14]. It can balance the regret value and the group utility value, and then select a compromise solution that meets the needs of decision-makers. The original VIKOR method was mainly used to solve multi-attribute decision-making with conflicts and incommensurability, and subsequent research was applied to GDM problems. This study mainly focuses on the GDM problem with fuzzy information based on VIKOR. The VIKOR method is a commonly used decision-making method in GDM problems. Recently, the VIKOR method has attracted the attention of many scholars [15,16]. For example, some researchers have introduced some VIKOR-based GDM methods in the intuitionistic fuzzy environment [17–19].

Scholars [20–22] proposed VIKOR-based GDM methods in the interval type-2 fuzzy environment. VIKOR-based GDM methods based on linguistic information were developed by some scholars [23–25]. Some researchers have subsequently proposed VIKOR-based GDM methods in various fuzzy information environments, [25] including intuitionistic fuzzy preference relations [26], interval-valued intuitionistic multiplicative preference relations [27], picture fuzzy sets [28], double hesitant fuzzy sets [29,30], and triangular fuzzy numbers [31]. Table 1 summarizes the research on VIKOR-based GDM methods with fuzzy information.

**Table 1:** VIKOR-based GDM methods with fuzzy information.

Researcher	Decision information	Application
Çalı and Balaman [17]	Intuitionistic fuzzy	Supplier selection
Salimian and Mousavi [18]	IVIF	COVID-19 risk assessment
Büyüközkan et al. [19]	IVIF	Airline Digital Capability Assessment
Wu et al. [20]	Interval type-2 fuzzy	Green supplier selection
Jana et al. [21]	Interval type-2 fuzzy	Evaluation of new product ideas
Meniz and Özkan [22]	Interval type-2 fuzzy	Vaccine selection for COVID-19
Wu et al. [23]	Linguistic information	Machine tool selection
Wu et al. [24]	Linguistic information	Assessment of the medical waste management system
Xu et al. [25]	Linguistic information	The risk evaluation of foreign direct investmen
Wan et al. [26]	Intuitionistic fuzzy preference relations	Selection of flexible manufacturing system
Lu et al. [27]	interval-valued intuitionistic multiplicative	Solid waste treatment technology selection
Singh and Kumar [28]	Picture fuzzy set	Supplier selection
Bashir et al. [29]	Dual hesitant fuzzy	Analyzing the impact factors of haze weather
Zhu et al. [30]	Dual hesitant fuzzy	Urban emergency capability assessment
		
Cheng et al. [31]	Triangular fuzzy	Supplier selection

By extending the VIKOR method to various fuzzy information environments, the research on VIKOR-based group decision-making methods has been further enriched, thereby expanding its scope of application. However, as described in **Research Question 2**, this study found that the existing research on the VIKOR method based on IVIF lacks a specific regret matrix, which carries group regret information. This is a limitation. If we can provide more reference information (characterized by specific vectors or matrices, etc.), then we can use them to handle more complex decision-making problems. In order to develop more information in the VIKOR-based GDM method, this paper attempts to provide a specific regret matrix for the VIKOR-based GDM model, thereby enhancing the comprehensive ability of the VIKOR method.

Research on decision-making methods based on projection measures mainly includes two aspects: (1) Decision-making methods based on classical projection measures. For example, Xu and Hu [11] established a projection model for IF-MADM. Xu and Liu [32] improved the traditional projection measure. Additionally, some scholars have applied classical projection measures to establish MADM models in interval fuzzy, IVIF, picture fuzzy, and linguistic fuzzy environments [33–36]. (2) Decision-making methods based on normalized projection measures. To address the shortcomings of classical projection measures, researchers have proposed normalized projection measures, which have become a key research focus. For instance, a VIKOR method based on picture fuzzy normalized projection [12] and a GDM method using normalized projection measures for hybrid information [37,38] have been proposed. Other researchers have introduced GDM modeling methods based on spherical fuzzy and Pythagorean fuzzy normalized projection measures [39,40]. To overcome the limitations of integrating traditional projection measures into the GDM and TOPSIS frameworks, Yue [41–43] contributed several GDM methods based on normalized projection measures for software quality evaluation.

The research summary of decision-making methods based on projection measures is shown in Table 2. These studies have further enriched and improved the GDM methods based on VIKOR technology and provided many important insights for decision science. However, as described in **Research Question 4**, the classical projection measure [11] and the normalized projection measures in the existing literature [41] still have defects, and new projection measure formulas need to be further proposed to enhance the comprehensive ability of the VIKOR method. To solve this problem, this study aims to develop a new normalized projection measure for two IVIF number vectors or matrices.

**Table 2:** Researches in the context of projection measures.

Researcher	Decision information	projection measures	Methodology	Application
Xu and Hu [11]	Intuitionistic fuzzy	Classical projection	MADM	Car Purchase Selection Decision
Xu and Liu [32]	Interval multiplicative preference	Improved classical projection	GDM	The partner selection decision
Pan and Wang [33]	Interval type fuzzy	Classical projection	TOPSIS	Renewable Energy Resources Selection
Tsao and Chen [34]	IVIF	Classical projection	MADM	Environmental watershed plan
Wei et al. [35]	Picture fuzzy set	Classical projection	MADM	Potential evaluation of emerging
Wu et al. [36]	Linguistic variable	Classical projection	MADM	Hospital decision support system
Wang et al. [12]	Picture fuzzy set	Normalized projection	VIKOR,MADM	Risk evaluation
Yue and Jia [34,37]	Real number and interval data	Normalized projection	GDM	Partner selection
Jia [38]	Hybrid decision information	Normalized projection	GDM	Marine equipment reliability assessment
Li [39]	Spherical fuzzy environment	Normalized projection	GDM	Community epidemic prevention management
Aldring [40]	Complex Pythagorean fuzzy	Normalized projection	MCGDM	Frequency identification
Yue [41]	Intuitionistic fuzzy information	Normalized projection	GDM	The satisfaction of smartphone users
Yue [42]	IVIF	Normalized projection	GDM	Software quality evaluation
Yue [43]	Interval information	Normalized projection	VIKOR,GDM	Evaluate Software quality

## 3. Preliminaries

### 3.1 Interval-valued intuitionistic Fuzzy set

**Definition 1.** If X is a non-empty set. $\tilde{A}$ is called IVIFs as follows [9]:


$$\tilde{A}=\left\{(x,\mu_{\tilde{A}}(x),\nu_{\tilde{A}}(x)\mathrm{\backslash rightleft}|x\in X\right\}$$ (1)

where $\mu_{\tilde{A}}(x)$ and $\nu_{\tilde{A}}(x)$ represent the membership and non-membership of elements belonging to X, and both are interval numbers: $\mu_{\tilde{A}}(x)=\left[\mu_{\tilde{A}}^{l}(x),\mu_{\tilde{A}}^{u}(x)\right]\subseteq [0,1]$, $\nu_{\tilde{A}}(x)=\left[\nu_{\tilde{A}}^{l}(x),\nu_{\tilde{A}}^{u}(x)\right]\subseteq [0,1]$, where $\mu_{\tilde{A}}^{u}(x)=\sup\mu_{\tilde{A}}(x)$,$\mu_{\tilde{A}}^{l}(x)=\inf\mu_{\tilde{A}}(x)$, $\nu_{\tilde{A}}^{u}(x)=\sup\nu_{\tilde{A}}(x)$, $\nu_{\tilde{A}}^{l}(x)=inf\nu (x)$, and satisfy $\mu_{\tilde{A}}^{u}(x)+\nu_{\tilde{A}}^{u}(x)\leq 1$. The degree of the hesitancy of elements in X can be expressed as $\pi_{\tilde{A}}(x)=\left[\pi_{\tilde{A}}^{l}(x),\pi_{\tilde{A}}^{u}(x)\right]$, and satisfies $\pi_{\tilde{A}}^{l}(x)=1-\mu_{\tilde{A}}^{u}(x)-\nu_{\tilde{A}}^{u}(x)$, $\pi_{\tilde{A}}^{u}(x)=1-\mu_{\tilde{A}}^{l}(x)-\nu_{\tilde{A}}^{l}(x)$. When $\mu_{\tilde{A}}(x)=\mu_{\tilde{A}}^{l}(x)=\mu_{\tilde{A}}^{u}(x)$, $\nu_{\tilde{A}}(x)=\nu_{\tilde{A}}^{l}(x)=\nu_{\tilde{A}}^{u}(x)$, it degenerates into intuitionistic fuzzy set. In reference [9], the following is referred to as IVIF number:


$$\tilde{\alpha }=\left(\left[\mu^{l},\mu^{u}\right]\right),\left[\nu^{l},\nu^{u}\right],$$ (2)

where $\left[\mu^{l},\mu^{u}\right]\subseteq [0,1],\left[\nu^{l},\nu^{u}\right]\subseteq [0,1]$ and ${0\leq \mu }^{u}+\nu^{u}\leq 1$, $\pi^{l}=1-\mu^{u}-\nu^{u}$, $\pi^{u}=1-\mu^{l}-\nu^{l}$.

**Definition 2.** [9] Let $\tilde{\alpha }=\left(\left[\mu^{l},\mu^{u}\right],\left[\nu^{l},\nu^{u}\right]\right)$, ${\tilde{\alpha }}_{1}=\left(\left[\mu_{1}^{l},\mu_{1}^{u}\right],\left[\nu_{1}^{l},\nu_{1}^{u}\right]\right)$, and ${\tilde{\alpha }}_{2}=\left(\left[\mu_{2}^{l},\mu_{2}^{u}\right],\left[\nu_{2}^{l},\nu_{2}^{u}\right]\right)$ be any three IVIF numbers, and define the following operation rules:

(1) ${\tilde{\alpha }}^{c}=\left(\left[\nu^{l},\nu^{u}\right],\left[\mu^{l},\mu^{u}\right]\right)$;
(2) $\lambda \tilde{\alpha }=\left(\left[{1-\left(1-\mu^{l}\right)}^{\lambda },{1-\left(1-\mu^{u}\right)}^{\lambda }\right],\left[{\left(\nu^{l}\right)}^{\lambda },{\left(\nu^{u}\right)}^{\lambda }\right]\right),\lambda >0;$
(3) ${\tilde{\alpha }}_{1}⨁{\tilde{\alpha }}_{2}=\left(\left[\mu_{1}^{l}+\mu_{2}^{l}-\mu_{1}^{l}\mu_{2}^{l},\mu_{1}^{u}+\mu_{2}^{u}-\mu_{1}^{u}\mu_{2}^{u}\right],\left[\nu_{1}^{l}\nu_{2}^{l},\nu_{1}^{u}\nu_{2}^{u}\right]\right);$
(4) ${\tilde{\alpha }}_{1}⨂{\tilde{\alpha }}_{2}=\left(\left[\mu_{1}^{l}u_{2}^{l},\mu_{1}^{u}\mu_{2}^{u}\right],\left[\nu_{1}^{l}+\nu_{2}^{l}-\nu_{1}^{l}\nu_{2}^{l},\nu_{1}^{u}+\nu_{2}^{u}-\nu_{1}^{u}\nu_{2}^{u}\right]\right).$

Subsequently, grounded on the existing operational rules of the IFS theory [44,45], we put forward the definitions of the subtraction and division operations of IVIF numbers. These definitions not merely inherit the fundamental properties of the operations of IFS, but also incorporate the characteristics of interval numbers, guaranteeing the closure and rationality of the operations.

**Definition 3.** Let ${\tilde{\alpha }}_{1}=\left(\left[\mu_{1}^{l},\mu_{1}^{u}\right],\left[\nu_{1}^{l},\nu_{1}^{u}\right]\right)$, ${\tilde{\alpha }}_{2}=\left(\left[\mu_{2}^{l},\mu_{2}^{u}\right],\left[\nu_{2}^{l},\nu_{2}^{u}\right]\right)$ be any two IVIF numbers and define the following arithmetic operations:

(1) ${\tilde{\alpha }}_{1}-{\tilde{\alpha }}_{2}={\tilde{\alpha }}_{1}⨂{\left({\tilde{\alpha }}_{2}\right)}^{c}=\left(\left[\mu_{1}^{l}\nu_{2}^{l},\mu_{1}^{u}\nu_{2}^{u}\right],\left[\nu_{1}^{l}+\mu_{2}^{l}-\nu_{1}^{l}\mu_{2}^{l},\nu_{1}^{u}+\mu_{2}^{u}-\nu_{1}^{u}\mu_{2}^{u}\right]\right)$;
(2) ${\tilde{\alpha }}_{1}÷{\tilde{\alpha }}_{2}={\tilde{\alpha }}_{1}⨁{\left({\tilde{\alpha }}_{2}\right)}^{c}=\left(\left[\mu_{1}^{l}+\nu_{2}^{l}-\mu_{1}^{l}\nu_{2}^{l},\mu_{1}^{u}+\nu_{2}^{u}-\mu_{1}^{u}\nu_{2}^{u}\right],\left[\nu_{1}^{l}\mu_{2}^{l},\nu_{1}^{u}\mu_{2}^{u}\right]\right)$.

Both the subtraction and division operations are realized via the complement operation. They possess symmetry and comply with the basic properties of the operations of IFS. The results of these operations remain IVIF numbers, guaranteeing the closure of the operations. Meanwhile, they also meet the operational boundary conditions. For details, refer to Property 1.

**Property 1** Let $\tilde{\alpha }=\left(\left[\mu^{l},\mu^{u}\right],\left[\nu^{l},\nu^{u}\right]\right)$ be an IVIF number, then the following operational properties hold:

(1) $\tilde{\alpha }-\left([0,0],[1,1]\right)=\tilde{\alpha }$;
(2) $\tilde{\alpha }-\left([1,1],[0,0]\right)=\left([0,0],\left[\nu^{l},\nu^{u}\right]\right)$;
(3) $\tilde{\alpha }÷\left([1,1],[0,0]\right)=\tilde{\alpha }$;
(4) $\tilde{\alpha }÷\left([0,0],[1,1]\right)=\left(\left[\mu^{l},\mu^{u}\right],[0,0]\right)$.

The subtraction and division operations of the IVIF numbers defined herein are founded on the existing operational rules of the IFS theory and integrate the characteristics of interval numbers. These operational relationships are rational, comply with the fundamental properties of the IFS theory, and display the anticipated behavior under boundary conditions. References [44,45] furnish a theoretical foundation for these operations.

### 3.2 Projection measurement

**Definition 4.** Let $\tilde{\alpha }=\left({\tilde{\alpha }}_{1},{\tilde{\alpha }}_{2},\cdots ,{\tilde{\alpha }}_{n}\right)$ and $\tilde{\beta }=\left({\tilde{\beta }}_{1},{\tilde{\beta }}_{2},\cdots ,{\tilde{\beta }}_{n}\right)$ be two *n*-dimensional IVIF vectors, where ${\tilde{\alpha }}_{i}=\left(\left[\mu_{\tilde{\alpha }}^{il},\mu_{\tilde{\alpha }}^{iu}\right],\left[\nu_{\tilde{\alpha }}^{il},\nu_{\tilde{\alpha }}^{iu}\right]\right)$ and ${\tilde{\beta }}_{i}=\left(\left[\mu_{\tilde{\beta }}^{il},\mu_{\tilde{\beta }}^{iu}\right],\left[\nu_{\tilde{\beta }}^{il},\nu_{\tilde{\beta }}^{iu}\right]\right)$. Literature [11] defines the projection of IVIFV as follows:


$${Proj}_{\tilde{\beta }}\left(\tilde{\alpha }\right)=\frac{\tilde{\alpha }\tilde{\beta }}{\left|\tilde{\beta }\right|}$$ (3)

where $\tilde{\alpha }\tilde{\beta }=\sum_{i=1}^{n}\left(\mu_{\tilde{\alpha }}^{il}\mu_{\tilde{\beta }}^{il}+\mu_{\tilde{\alpha }}^{iu}\mu_{\tilde{\beta }}^{iu}+\nu_{\tilde{\alpha }}^{il}\nu_{\tilde{\beta }}^{il}+\nu_{\tilde{\alpha }}^{iu}\nu_{\tilde{\beta }}^{iu}+\pi_{\tilde{\alpha }}^{il}\pi_{\tilde{\beta }}^{il}+\pi_{\tilde{\alpha }}^{iu}\pi_{\tilde{\beta }}^{iu}\right)$,${\left|\tilde{\beta }\right|}^{2}=\sum_{i=1}^{n}\left({\left(\mu_{\tilde{\beta }}^{il}\right)}^{2}+{\left(\mu_{\tilde{\beta }}^{iu}\right)}^{2}+{\left(\nu_{\tilde{\beta }}^{il}\right)}^{2}+{\left(\nu_{\tilde{\beta }}^{iu}\right)}^{2}+{\left(\pi_{\tilde{\beta }}^{il}\right)}^{2}+{\left(\pi_{\tilde{\beta }}^{iu}\right)}^{2}\right)$, $\left|\tilde{\beta }\right|=\sqrt{{\left|\tilde{\beta }\right|}^{2}}$,$\pi_{\tilde{\alpha }}^{il}=1-\mu_{\tilde{\alpha }}^{iu}-\nu_{\tilde{\alpha }}^{iu}$,$\pi_{\tilde{\alpha }}^{iu}=1-\mu_{\tilde{\alpha }}^{il}-\nu_{\tilde{\alpha }}^{il}$,$\pi_{\tilde{\beta }}^{il}=1-\mu_{\tilde{\beta }}^{iu}-\nu_{\tilde{\beta }}^{iu}$, $\pi_{\tilde{\beta }}^{iu}=1-\mu_{\tilde{\beta }}^{il}-\nu_{\tilde{\beta }}^{il}$.

As shown by Definition 4, the larger the value of ${Proj}_{\tilde{\beta }}\left(\tilde{\alpha }\right)$, the closer the IVIF vector $\tilde{\alpha }$ is to the $\tilde{\beta }$. it is clear from the analysis that Eq. (3) is not always reasonable. As shown in Example 1 below.

**Example 1.** Let $\tilde{\alpha }=\left(\left([0.8,0.9],[0,0.1]\right),\left([0.89,0.9],[0,0.1]\right)\right)$ and $\tilde{\beta }=\left(\left([0.7,0.8],[0,0.2]\right),\left([0.6,0.7],[0,0.2]\right)\right)$, calculate ${Proj}_{\tilde{\beta }}\left(\tilde{\beta }\right)\approx 1.523$ and ${Proj}_{\tilde{\beta }}\left(\tilde{\alpha }\right)=1.699$ according to Eq. (3). Obviously, ${Proj}_{\tilde{\beta }}\left(\tilde{\alpha }\right)>{Proj}_{\tilde{\beta }}\left(\tilde{\beta }\right)$, this is contradictory. And the projected value of Eq. (3) does not satisfy the normalization condition: the ${0\leq Proj}_{\tilde{\beta }}\left(\tilde{\alpha }\right)\leq 1$. The projected values may sometimes be very large and sometimes very small, which is not conducive to decision-making by experts, which is also a drawback.

To address the above shortcomings of the classical projection measure, some researchers [41] proposed the following normalized projection measure formula:


$${NProj}_{\tilde{\beta }}\left(\tilde{\alpha }\right)=\frac{\tilde{\alpha }\tilde{\beta }}{\tilde{\alpha }\tilde{\beta }+\left|{\left|\tilde{\alpha }\right|}^{2}-{\left|\tilde{\beta }\right|}^{2}\right|}$$ (4)

where $\left|\tilde{\alpha }\right|=\sqrt{{\left|\tilde{\alpha }\right|}^{2}}$,${\left|\tilde{\alpha }\right|}^{2}=\sum_{i=1}^{n}\left({\left(\mu_{\tilde{\alpha }}^{il}\right)}^{2}+{\left(\mu_{\tilde{\alpha }}^{iu}\right)}^{2}+{\left(\nu_{\tilde{\alpha }}^{il}\right)}^{2}+{\left(\nu_{\tilde{\alpha }}^{iu}\right)}^{2}+{\left(\pi_{\tilde{\alpha }}^{il}\right)}^{2}+{\left(\pi_{\tilde{\alpha }}^{iu}\right)}^{2}\right)$, others are the same as in Eq. (3) above.

Usually the closer the value of Eq. (4) is to 1, the closer the IVIF vector $\tilde{\alpha }$ is to the vector $\tilde{\beta }$, however, this study found that Eq. (4) still has a hidden flaw: when $\tilde{\alpha }=\tilde{\beta }$, there is ${NProj}_{\tilde{\beta }}\left(\tilde{\alpha }\right)=1$ in all cases, see Example 2 below.

**Example 2.** Let $\tilde{\alpha }=\left(\left([0.3,0.5],[0.2,0.4]\right),\left([0.4,0.6],[0.3,0.4]\right)\right)$ and $\tilde{\beta }=\left(\left([0.2,0.4],[0.3,0.5]\right),\left([0.3,0.4],[0.4,0.6]\right)\right)$, using Eq. (4), compute ${NProj}_{\tilde{\beta }}\left(\tilde{\alpha }\right)=1$, but obviously $\tilde{\alpha }\neq \tilde{\beta }$, which is a contradiction.

Also Eq. (4) is still a bi-directional projection which satisfies satisfies ${NProj}_{\tilde{\beta }}\left(\tilde{\alpha }\right)={NProj}_{\tilde{\alpha }}\left(\tilde{\beta }\right)$ and bi-directional projection has excellent performance. But few satisfy this condition, so bidirectional projection is a limitation. Therefore unidirectional projection is more realistic.

To address the shortcomings and limitations of the above projection measure, we give a new normalized IVIF vector projection measure formula:

**Definition 5.** Let $\tilde{\alpha }=\left({\tilde{\alpha }}_{1},{\tilde{\alpha }}_{2},\cdots ,{\tilde{\alpha }}_{n}\right)$ and $\tilde{\beta }=\left({\tilde{\beta }}_{1},{\tilde{\beta }}_{2},\cdots ,{\tilde{\beta }}_{n}\right)$ be two *n*-dimensional IVIF vectors and define the normalized projection of the IVIF vector $\tilde{\alpha }$ on the $\tilde{\beta }$ as:


$${NProj}_{\tilde{\beta }}\left(\tilde{\alpha }\right)=\frac{\tilde{\alpha }\tilde{\beta }}{\left|\tilde{\alpha }\right|\left|\tilde{\beta }\right|+\left|\tilde{\alpha }\tilde{\beta }-{\left|\tilde{\beta }\right|}^{2}\right|+\left|{\left|\tilde{\alpha }\right|}^{2}-{\left|\tilde{\beta }\right|}^{2}\right|}$$ (5)

Eq. (5) satisfies the normalization condition: ${0\leq Proj}_{\tilde{\beta }}\left(\tilde{\alpha }\right)\leq 1$.

The closer the value of ${NProj}_{\tilde{\beta }}\left(\tilde{\alpha }\right)$ in Eq. (5) is to 1, the closer the $\tilde{\alpha }$ is to $\tilde{\beta }$. Using Eq. (5) to calculate **Example 1**, it can get $N{Proj}_{\tilde{\beta }}\left(\tilde{\beta }\right)=1$, ${NProj}_{\tilde{\beta }}\left(\tilde{\alpha }\right)\approx 0.687$, and obviously ${NProj}_{\tilde{\beta }}\left(\tilde{\alpha }\right)<N{Proj}_{\tilde{\beta }}\left(\tilde{\beta }\right)$. From Eq. (5) to calculate **Example 2**, it can get ${NProj}_{\tilde{\beta }}\left(\tilde{\alpha }\right)\approx 0.919\neq 1$, that Eq. (5) is more perfect than Eqs. (3) and (4), and it solves the above problems and limitations. Therefore, Eq. (5) can be more accurate to measure the proximity of two IVIF vectors.

We extend the normalized projection measure formulation to the two high-dimensional IVIF matrices below:

**Definition 6**. Let $\tilde{A}={\left({\tilde{a}}_{kj}\right)}_{t\times n}$ and $\tilde{B}={\left({\tilde{b}}_{kj}\right)}_{t\times n}$ be two IVIF matrices, where ${\tilde{a}}_{kj}=\left(\left[\mu_{kj}^{l},\mu_{kj}^{u}\right],\left[\nu_{kj}^{l},\nu_{kj}^{u}\right]\right)$, ${\tilde{b}}_{kj}=\left(\left[\tau_{kj}^{l},\tau_{kj}^{u}\right],\left[\upsilon_{kj}^{l},\upsilon_{kj}^{u}\right]\right)$, then define the generalized normalized projection of the IVIF matrix $\tilde{A}$ on the $\tilde{B}$ as:


$${GNProj}_{\tilde{B}}\left(\tilde{A}\right)=\frac{\tilde{A}\tilde{B}}{\left|\tilde{A}\right|\left|\tilde{B}\right|+\left|\tilde{A}\tilde{B}-{\left|\tilde{B}\right|}^{2}\right|+\left|{\left|\tilde{A}\right|}^{2}-{\left|\tilde{B}\right|}^{2}\right|}$$ (6)

where $\tilde{A}\tilde{B}=\sum_{k=1}^{t}\sum_{j=1}^{n}\left(\mu_{kj}^{l}\tau_{kj}^{l}+\mu_{kj}^{u}\tau_{kj}^{u}+\nu_{kj}^{l}\upsilon_{kj}^{l}+\nu_{kj}^{u}\upsilon_{kj}^{u}+\pi_{kj}^{l}\eta_{kj}^{l}+\pi_{kj}^{u}\eta_{kj}^{u}\right)$*,* $\left|\tilde{A}\right|=\sqrt{{\left|\tilde{A}\right|}^{2}}$*,*${\left|\tilde{A}\right|}^{2}=\sum_{k=1}^{t}\sum_{j=1}^{n}\left({\left(\mu_{kj}^{l}\right)}^{2}+{\left(\mu_{kj}^{u}\right)}^{2}+{\left(\nu_{kj}^{l}\right)}^{2}+{\left(\nu_{kj}^{u}\right)}^{2}+{\left(\pi_{kj}^{l}\right)}^{2}+{\left(\pi_{kj}^{u}\right)}^{2}\right)$*,*$\left|\tilde{B}\right|=\sqrt{{\left|\tilde{B}\right|}^{2}},{\left|\tilde{B}\right|}^{2}=\sum_{k=1}^{t}\sum_{j=1}^{n}\left({\left(\tau_{kj}^{l}\right)}^{2}+{\left(\tau_{kj}^{u}\right)}^{2}+{\left(\upsilon_{kj}^{l}\right)}^{2}+{\left(\upsilon_{kj}^{u}\right)}^{2}+{\left(\eta_{kj}^{l}\right)}^{2}+{\left(\eta_{kj}^{u}\right)}^{2}\right)$, and $\pi_{kj}^{l}=1-\mu_{kj}^{u}-\nu_{kj}^{u}$*,*$\pi_{kj}^{u}=1-\mu_{kj}^{l}-\nu_{kj}^{l}$*,*$\eta_{kj}^{l}=1-\tau_{kj}^{u}-\upsilon_{kj}^{u}$*,*$\eta_{kj}^{u}=1-\tau_{kj}^{l}-\upsilon_{kj}^{l}$*.*

The closer the value of ${GNProj}_{\tilde{B}}\left(\tilde{A}\right)$ in Eq. (6) is to 1, the closer the IVIF matrix $\tilde{A}$ is to $\tilde{B}$. Therefore, Eq. (6) can be used to measure the proximity of two IVIF matrices.

## 4. GDM method establishment

In this study, a new GDM method is established using the developed projection measure formulation fused with the VIKOR technique in the IVIF environment.

For convenience, let M={1,2,⋯,m}, N={1,2,⋯,n} and T={1,2,⋯,t}. Suppose that $A=\left\{A_{i}|i\in M\right\}$ is a set of feasible alternatives, $D=\left\{d_{k}|k\in T\right\}$ is a group of experts, and $w=\left(w_{1},w_{2},\cdots ,w_{n}\right)$ is the weight vector of the attribute, where $\sum_{j=1}^{n}w_{j}=1$. Suppose there is a real decision-making case with m alternatives. Decision-making experts from different fields are invited to form an expert group for decision-making, and each field consists of more than one decision-making expert, and in this study we assume that the evaluation value of experts in the same field has already been assembled, and is finally indicated by one expert. The expert group determines the evaluation attribute set of the decision problem, which is denoted as $G=\left\{g_{1,}g_{2},\cdots ,g_{n}\right\}$. multiple experts in each field evaluate and score the alternatives of the decision problem separately, and the evaluation values are expressed as IVIF numbers. The scores of the experts in the same domain are then pooled, and the pooled evaluation values are still IVIF numbers. The final evaluation information for each alternative is pooled into an IVIF evaluation matrix according to the t domains, denoted as:


$$\begin{array}{c} {\tilde{A}}_{i}=\begin{array}{c} A_{1}\\ \vdots \\ A_{t} \end{array}(\begin{array}{ccc} {\tilde{a}}_{11}^{i} & \cdots & {\tilde{a}}_{1n}^{i}\\ \vdots & \ddots & \vdots \\ {\tilde{a}}_{t1}^{i} & \cdots & {\tilde{a}}_{tn}^{i} \end{array}),i\in M \end{array}$$ (7)

where ${\tilde{A}}_{i}={\left({\tilde{a}}_{kj}^{i}\right)}_{t\times n}$ denotes the information matrix of the evaluation of alternative $A_{i}$ by the group of decision-making experts, and ${\tilde{a}}_{kj}^{i}$ denotes the evaluation value $g_{j}$ of the *j-*th attribute of the *i*-th alternative $A_{i}$ by the *k*-th group of domain experts, ${\tilde{a}}_{kj}^{i}$ is an IVIF number, and it is clear that ${\tilde{A}}_{i}$ is a normalized matrix.

The attribute weight vector $w=\left\{w_{j}|j\in N\right\}$ is determined by the expert community and the weighted evaluation matrix is computed denoted as:


$${\tilde{B}}_{i}={\left({\tilde{b}}_{kj}^{i}\right)}_{t\times n},i\in M$$ (8)

where ${\tilde{b}}_{kj}^{i}=\omega_{j}{\tilde{a}}_{kj}^{i}=\left(\left[\tau_{kj}^{il},\tau_{kj}^{iu}\right],\left[\left[\upsilon_{kj}^{il},\upsilon_{kj}^{iu}\right]\right]\right)$*,* and $\tau_{kj}^{il}=1-{\left(1-\mu_{kj}^{il}\right)}^{\omega_{j}}$*,* $\tau_{kj}^{iu}=1-{\left(1-\mu_{kj}^{iu}\right)}^{\omega_{j}}$*,* $\varphi_{ij}^{kl}={\left(\nu_{ij}^{kl}\right)}^{\omega_{j}}$*,* $\upsilon_{kj}^{iu}={\left(\nu_{kj}^{iu}\right)}^{\omega_{j}}$*.*

Based on the VIKOR technique, determine the positive ideal evaluation matrix, assuming all attribute indicators are benefit-oriented.


$${\tilde{B}}^{+}={\left({\tilde{b}}_{kj}^{+}\right)}_{t\times n}$$ (9)

where ${\tilde{b}}_{kj}^{+}=\left(\left[\tau_{kj}^{+l},\tau_{kj}^{+u}\right],\left[\upsilon_{kj}^{+l},\upsilon_{kj}^{+u}\right]\right)=\left(\left[\max_{i}\tau_{kj}^{il},\max_{i}\tau_{kj}^{iu}\right],\left[\left[\min_{i}\upsilon_{kj}^{il},\min_{i}\upsilon_{kj}^{iu}\right]\right]\right)$.

The proximity of the weighted evaluation matrix ${\tilde{B}}_{i}$ to the positive ideal evaluation matrix ${\tilde{B}}^{+}$ is calculated separately for each alternative using Eq. (6):


$${{GN}_{i}=GNProj}_{{\tilde{B}}^{+}}\left({\tilde{B}}_{i}\right),i\in M$$ (10)

where ${GNProj}_{{\tilde{B}}^{+}}\left({\tilde{B}}_{i}\right)=\frac{{\tilde{B}}_{i}{\tilde{B}}^{+}}{\left(\left|{\tilde{B}}_{i}\right|\left|{\tilde{B}}^{+}\right|+\left|{\tilde{B}}_{i}{\tilde{B}}^{+}-{\left|{\tilde{B}}^{+}\right|}^{2}\right|+\left|{\left|{\tilde{B}}_{i}\right|}^{2}-{\left|{\tilde{B}}^{+}\right|}^{2}\right|\right)}$.

We refer to ${GN}_{i}$ (i=1,2,⋯,m) as the group utility value, let ${GN}^{+}=\max_{1\leq i\leq m}\left\{{GN}_{i}\right\}$, ${GN}^{-}=\min_{1\leq i\leq m}\left\{{GN}_{i}\right\}$, ${GN}^{+}$ is called the largest group utility value and ${GN}^{-}$ is called the smallest group utility value, the normalized group utility value of each alternative $A_{i}$ is defined as:


$${GNC}_{i}=\left\{ \begin{array}{c} \frac{{GN}_{i}-{GN}^{-}}{{{GN}^{+}-GN}^{-}},if{GN}^{+}\neq {GN}^{-}\\ 1,if{GN}^{+}={GN}^{-} \end{array}\right.,i\in M$$ (11)

where a larger value indicates a better $A_{i}$ alternative.

In the VIKOR technique, a specific regret matrix is not provided. The regret matrix is a crucial component that indicates the difference between the evaluation matrix of the current alternative and the positive ideal evaluation matrix. In this study, the evaluation matrix and positive ideal evaluation matrix of each alternative are represented as IVIF numbers. To calculate the regret matrix of each alternative separately, we use the subtraction operation rules of the two matrices and the subtraction operation rules of the two IVIF numbers in Definition 1. The calculation is noted as follows:


$${\tilde{R}}_{i}={\left({\tilde{r}}_{kj}^{i}\right)}_{t\times n},i\in M$$ (12)

where ${\tilde{r}}_{kj}^{i}={{\tilde{b}}_{kj}^{i}-\tilde{b}}_{kj}^{+}=\left(\left[\varphi_{kj}^{il},\varphi_{kj}^{iu}\right],\left[\left[\phi_{kj}^{il},\phi_{kj}^{iu}\right]\right]\right)$,${\tilde{b}}_{kj}^{i}$ and ${\tilde{b}}_{kj}^{+}$ are the same as in Eqs. (8) and (9). From (1) in Definition 3, we calculate $\varphi_{kj}^{il}=\tau_{kj}^{il}\upsilon_{kj}^{+l}$,$\varphi_{kj}^{iu}=\tau_{kj}^{iu}\upsilon_{kj}^{+u}$, $\phi_{kj}^{il}=\upsilon_{kj}^{il}{+\tau_{kj}^{+l}-\upsilon_{kj}^{il}\tau }_{kj}^{+l}$, $\phi_{kj}^{iu}=\upsilon_{kj}^{iu}{+\tau_{kj}^{+u}-\upsilon_{kj}^{iu}\tau }_{kj}^{+u}$.

Based on the definition of the regret matrix, we define the smallest individual regret matrix as:


$${\tilde{R}}^{+}={\left({\tilde{r}}_{kj}^{+}\right)}_{t\times n}$$ (13)

where ${\tilde{r}}_{kj}^{+}=\left(\left[\varphi_{kj}^{+l},\varphi_{kj}^{+u}\right],\left[\phi_{kj}^{+l},\phi_{kj}^{+u}\right]\right)=\left(\left[\min_{i}\varphi_{kj}^{il},\min_{i}\varphi_{kj}^{iu}\right],\left[\left[\max_{i}\phi_{kj}^{il},\max_{i}\phi_{kj}^{iu}\right]\right]\right)$.

Then the proximity of the individual regret matrix ${\tilde{R}}_{i}$ to the smallest individual regret matrix ${\tilde{R}}^{+}$ is calculated separately for each alternative using Eq. (6):


$${{GR}_{i}=GNProj}_{{\tilde{R}}^{+}}\left({\tilde{R}}_{i}\right),i\in M$$ (14)

where ${GNProj}_{{\tilde{R}}^{+}}\left({\tilde{R}}_{i}\right)=\frac{{\tilde{R}}_{i}{\tilde{R}}^{+}}{\left(\left|{\tilde{R}}_{i}\right|\left|{\tilde{R}}^{+}\right|+\left|{\tilde{R}}_{i}{\tilde{R}}^{+}-{\left|{\tilde{R}}^{+}\right|}^{2}\right|+\left|{\left|\tilde{R}\right|}^{2}-{\left|{\tilde{R}}^{+}\right|}^{2}\right|\right)}$.

We call ${GR}_{i}$ the group regret value and let ${GR}^{+}=\max_{1\leq i\leq m}\left\{{GR}_{i}\right\}$, ${GR}^{-}=\min_{1\leq i\leq m}\left\{{GR}_{i}\right\}$, where ${GR}^{+}$ is called the largest individual regret value and ${GR}^{-}$ is called the smallest individual regret value, then the normalized individual regret value of alternative $A_{i}$ is defined as:


$${GRC}_{i}=\left\{ \begin{array}{c} \frac{{GR}_{i}-{GR}^{-}}{{{GR}^{+}-GR}^{-}},if{GR}^{+}\neq {GR}^{-}\\ 1,if{GR}^{+}={GR}^{-} \end{array}\right.,i\in M$$ (15)

where a larger value indicates a better alternative $A_{i}$.

The comprehensive measure based on the VIKOR technique is defined as:


$${Q_{i}=\alpha {GNC}_{i}+(1-\alpha )GRC}_{i},i\in M$$ (16)

where α is called the decision-making mechanism coefficient, which satisfies α∈[0,1]. When α>0.5, the decision-maker prefers to make decisions based on group utility; when α<0.5, the decision-maker prefers to make decisions based on group regret; and when α=0.5, indicates that decision-makers take a balanced and eclectic approach when making decisions.. α=0.5 is generally used.

Let the result of the ranking by the comprehensive measure $Q_{i}$ (i=1,2,⋯,m) be $A^{\left(1\right)}>A^{\left(2\right)}>\cdots A^{\left(J\right)}\cdots >A^{\left(m\right)}$. The next step involves ranking the alternatives based on the following two conditions:

**Condition 1** Acceptable dominance condition: $Q\left(A^{\left(1\right)}\right)-Q\left(A^{\left(2\right)}\right)\geq \frac{1}{\left(m-1\right)}$.

**Condition 2** Acceptable stability condition in the decision-making process: solution $A^{\left(1\right)}$ must also be the first solution in the ranking according to ${GNC}_{i}$ or ${GRC}_{i}$.

Alternative $A^{\left(1\right)}$ is the most stable and optimal choice in the decision-making process when both Ranking Condition 1 and Ranking Condition 2 are met. If the ranking condition 2 is not satisfied, both scheme $A^{\left(1\right)}$ and scheme $A^{\left(2\right)}$ are compromise solution solutions, at this time, you can continue to determine the largest J by $Q\left(A^{\left(1\right)}\right)-Q\left(A^{\left(2\right)}\right)<\frac{1}{\left(m-1\right)}$, at this time, $A^{\left(1\right)},A^{\left(2\right)}\cdots A^{\left(J\right)}$ are both compromise solutions.

## 5. Procedure for proposed methodology

**Fig 1:**
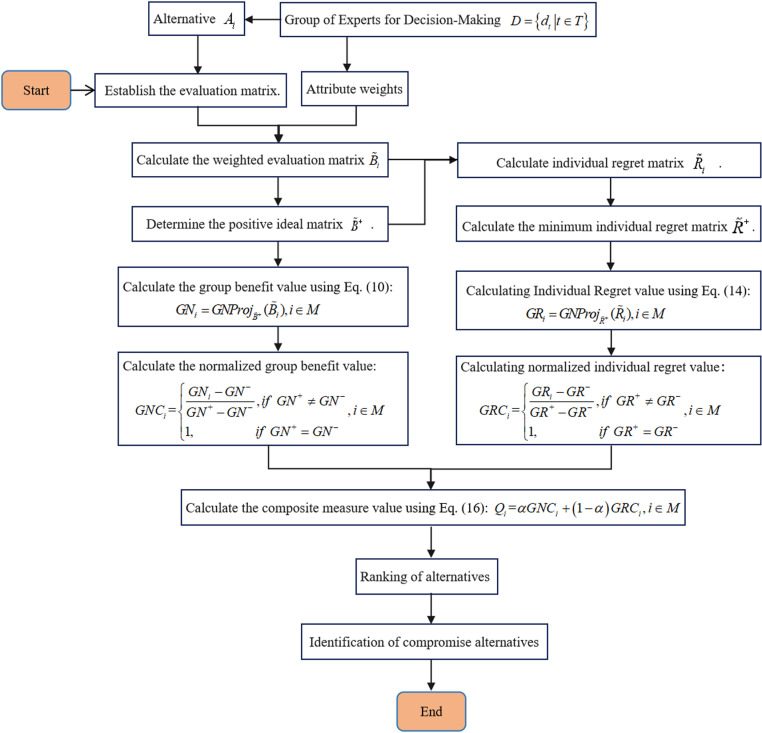
Flowchart of the GDM method in this paper.

**Fig 1** is the flowchart of the proposed method of this paper.The following are the basic steps for implementing the GDM method as outlined in this paper:

**Table:** 

Step 1. Establish the evaluation matrix. Based on Eq. (7), establish the evaluation matrix ( ${\tilde{A}}_{i}$ ) of the decision-making expert group for each alternative. Step 2. Calculate the weighted evaluation matrix. Let the attribute weight vector determined by the decision-making expert group is w , then calculate the weighted evaluation matrix ( ${\tilde{B}}_{i}$ ) of each alternative based on Eq. (8).
**Step 3.** Determine the positive ideal evaluation matrix of GDM. Determine the positive ideal evaluation matrix ( ${\tilde{B}}^{+}$ ) based on Eq. (9).
**Step 4.** Calculate the group utility value. Calculate the group utility value ( ${GN}_{i}$ ) of each alternative based on Eq. (10).
**Step 5.** Calculate the normalized group utility value. Calculate the normalized group utility value ( ${GNC}_{i}$ ) for each alternative according to Eq. (11).
**Step 6.** Calculate individual regret matrix. Calculate the individual regret matrix ( ${\tilde{R}}_{i}$ ) for each alternative selection according to Eq. (12).
**Step 7.** Calculate the minimum individual regret matrix. Calculate the minimum individual regret matrix ( ${\tilde{R}}^{+}$ ) according to Eq. (13).
**Step 8.** Calculating Individual Regrets. Calculating Individual Regret value ( ${GR}_{i}$ ) for each alternative according to Eq. (14).
**Step 9.** Calculating normalized individual regret values. Calculating normalized individual regret value ( ${GRC}_{i}$ ) for each alternative selection according to Eq. (15).
**Step 10.** Calculate the comprehensive measure value Based on the VIKOR technology idea, determine the decision mechanism coefficient α according to the preference of decision-making experts, and then calculate the comprehensive measure value ( $Q_{i}$ ) of each alternative using Eq. (16).
**Step 11.** Ranking of alternatives. Sort the alternatives according to the comprehensive measure value ( $Q_{i}$ ) from the largest to the smallest, and finally determine the final ranking results according to the ranking conditions of VIKOR technology.
**Step 12.** Identification of compromise alternatives Determine the compromise program based on the compromise **condition 1** and **condition 2.**

## 6. Data experiment

In this section, an Illustrative example of enterprise patent quality assessment is used to verify the feasibility and effectiveness of the proposed method in this paper. The example data is based on the literature [46], and all calculations and experimental analyses are performed using MATLAB software. The experimental data and calculation details can be obtained from the following link: https://github.com/junxx12/matlab.git.

### 6.1 Illustrative example

This section aims to demonstrate the feasibility and effectiveness of the GDM method proposed in this paper for assessing patent quality using specific examples.

A company needs to purchase a patent for an important component of a product, and there are four alternative suppliers. The patents of the four alternative suppliers form a set represented by $\left\{A_{1},A_{2},A_{3},A_{4}\right\}$. Based on the literature [46], the quality of enterprise patents will be evaluated from three aspects: patent technical level, patent conversion utility, and patent protection regulations. The evaluation attributes are represented by $\left\{g_{1},g_{2},g_{3}\right\}$, and their attribute weights are determined as $\left(w_{1},w_{2},w_{3}\right)=(0.3,0.4,0.3)$ respectively after discussion by the expert group. The five expert groups from different fields and with rich experience are represented by the set $\left\{d_{1},d_{2},d_{3},d_{4},d_{5}\right\}$, and the evaluation values of each expert are all expressed in IVIF numbers. Now, the four patents $\left\{A_{1},A_{2},A_{3},A_{4}\right\}$ are evaluated using the method proposed in this paper.

According to Step 1 expert group evaluates the patent quality of each alternative supplier according to the attribute index, according to the evaluation information to establish the evaluation matrix (${\tilde{A}}_{i},i=1,2,3,4$) of the patent quality of each alternative supplier, specifically shown in Table 3.

**Table 3:** Evaluation matrix for each alternative.

Evaluation matrix	Experts	$g_{1}$	$g_{2}$	$g_{3}$
${\tilde{A}}_{1}$	$d_{1}$	([0.8,0.9],[0.1,0.1])	([0.3,0.4],[0.4,0.5])	([0.2,0.5],[0.3,0.5])
	$d_{2}$	([0.3,0.4],[0.4,0.6])	([0.5,0.6],[0.2,0.3])	([0.3,0.4],[0.4,0.5])
	$d_{3}$	([0.6,0.9],[0.1,0.1])	([0.7,0.8],[0.1,0.2])	([0.2,0.3],[0.5,0.6])
	$d_{4}$	([0.1,0.2],[0.8,0.8])	([0.6,0.8],[0.1,0.2])	([0.1,0.2],[0.5,0.8])
	$d_{5}$	([0.4,0.5],[0.2,0.4])	([0.1,0.2],[0.7,0.8])	([0.6,0.7],[0.1,0.2])
${\tilde{A}}_{2}$	$d_{1}$	([0.5,0.8],[0.1,0.2])	([0.4,0.5],[0.3,0.4])	([0.2,0.4],[0.4,0.5])
	$d_{2}$	([0.4,0.6],[0.3,0.4])	([0.5,0.6],[0.3,0.4])	([0.7,0.8],[0.1,0.2])
	$d_{3}$	([0.3,0.4],[0.4,0.6])	([0.1,0.2],[0.6,0.8])	([0.6,0.8],[0.1,0.2])
	$d_{4}$	([0.4,0.7],[0.1,0.2])	([0.7,0.8],[0.1,0.2])	([0.5,0.7],[0.1,0.2])
	$d_{5}$	([0.6,0.9],[0.1,0.1])	([0.2,0.4],[0.4,0.5])	([0.4,0.5],[0.3,0.4])
${\tilde{A}}_{3}$	$d_{1}$	([0.6,0.9],[0.1,0.1])	([0.2,0.4],[0.4,0.5])	([0.4,0.5],[0.3,0.4])
	$d_{2}$	([0.1,0.2],[0.7,0.8])	([0.9,0.9],[0.1,0.1])	([0.5,0.7],[0.1,0.3])
	$d_{3}$	([0.6,0.7],[0.2,0.3])	([0.6,0.8],[0.2,0.2])	([0.1,0.2],[0.5,0.6])
	$d_{4}$	([0.4,0.5],[0.4,0.5])	([0.5,0.6],[0.1,0.2])	([0.7,0.8],[0.1,0.2])
	$d_{5}$	([0.4,0.5],[0.3,0.4])	([0.3,0.4],[0.1,0.2])	([0.5,0.8],[0.1,0.2])
${\tilde{A}}_{4}$	$d_{1}$	([0.7,0.9],[0.1,0.1])	([0.1,0.2],[0.6,0.7])	([0.3,0.4],[0.5,0.6])
	$d_{2}$	([0.6,0.8],[0.1,0.2])	([0.6,0.8],[0.2,0.2])	([0.1,0.4],[0.5,0.6])
	$d_{3}$	([0.5,0.8],[0.1,0.2])	([0.7,0.8],[0.1,0.2])	([0.5,0.7],[0.1,0.3])
	$d_{4}$	([0.4,0.6],[0.3,0.4])	([0.4,0.5],[0.4,0.5])	([0.7,0.8],[0.1,0.2])
	$d_{5}$	([0.3,0.4],[0.1,0.2])	([0.1,0.3],[0.5,0.7])	([0.5,0.8],[0.1,0.2])

Based on Step 2, establish the attribute weighted evaluation matrix (${\tilde{B}}_{i}$), and the corresponding weighted evaluation matrices of the four alternatives are shown in Table 4.

**Table 4:** Weighted evaluation matrix for each alternative.

Evaluation matrCix	Experts	$g_{1}$	$g_{2}$	$g_{3}$
${\tilde{B}}_{1}$	$d_{1}$	([0.38,0.50],[0.50,0.50])	([0.13,0.18],[0.69,0.76])	([0.06,0.19],[0.70,0.81])
	$d_{2}$	([0.10,0.14],[0.76,0.86])	([0.24,0.31],[0.53,0.62])	([0.10,0.14],[0.76,0.81])
	$d_{3}$	([0.24,0.50],[0.50,0.50])	([0.38,0.47],[0.40,0.53])	([0.06,0.10],[0.81,0.86])
	$d_{4}$	([0.03,0.06],[0.94,0.94])	([0.31,0.47],[0.40,0.53])	([0.03,0.06],[0.81,0.94])
	$d_{5}$	([0.14,0.19],[0.62,0.76])	([0.04,0.09],[0.87,0.91])	([0.24,0.30],[0.50,0.62])
${\tilde{B}}_{2}$	$d_{1}$	([0.19,0.38],[0.50,0.62])	([0.18,0.24],[0.62,0.69])	([0.06,0.14],[0.76,0.81])
	$d_{2}$	([0.14,0.24],[0.70,0.76])	([0.24,0.31],[0.62,0.69])	([0.30,0.38],[0.50,0.62])
	$d_{3}$	([0.10,0.14],[0.76,0.86])	([0.04,0.09],[0.82,0.91])	([0.24,0.38],[0.50,0.62])
	$d_{4}$	([0.14,0.30],[0.50,0.62])	([0.38,0.47],[0.40,0.53])	([0.19,0.30],[0.50,0.62])
	$d_{5}$	([0.24,0.50],[0.50,0.50])	([0.09,0.18],[0.69,0.76])	([0.14,0.19],[0.70,0.76])
${\tilde{B}}_{3}$	$d_{1}$	([0.24,0.50],[0.50,0.50])	([0.09,0.18],[0.69,0.76])	([0.14,0.19],[0.70,0.76])
	$d_{2}$	([0.03,0.06],[0.90,0.94])	([0.60,0.60],[0.40,0.40])	([0.19,0.30],[0.50,0.70])
	$d_{3}$	([0.24,0.30],[0.62,0.70])	([0.31,0.47],[0.53,0.53])	([0.03,0.06],[0.81,0.86])
	$d_{4}$	([0.14,0.19],[0.76,0.81])	([0.24,0.31],[0.40,0.53])	([0.30,0.38],[0.50,0.62])
	$d_{5}$	([0.14,0.19],[0.70,0.76])	([0.13,0.18],[0.40,0.53])	([0.19,0.38],[0.50,0.62])
${\tilde{B}}_{4}$	$d_{1}$	([0.30,0.50],[0.50,0.50])	([0.04,0.09],[0.82,0.87])	([0.10,0.14],[0.81,0.86])
	$d_{2}$	([0.24,0.38],[0.50,0.62])	([0.31,0.47],[0.53,0.53])	([0.03,0.14],[0.81,0.86])
	$d_{3}$	([0.19,0.38],[0.50,0.62])	([0.38,0.47],[0.40,0.53])	([0.19,0.30],[0.50,0.70])
	$d_{4}$	([0.14,0.24],[0.70,0.76])	([0.18,0.24],[0.69,0.76])	([0.30,0.38],[0.50,0.62])
	$d_{5}$	([0.10,0.14],[0.50,0.62])	([0.04,0.13],[0.76,0.87])	([0.19,0.38],[0.50,0.62])

According to Step 3, determine the group utility positive ideal evaluation matrix (${\tilde{B}}^{+}$), and the calculation results are shown in Table 5 below.

**Table 5:** Positive Ideal Evaluation Matrix for Group utilitys.

Evaluation matrix	Experts	$g_{1}$	$g_{2}$	$g_{3}$
${\tilde{B}}^{+}$	$d_{1}$	([0.38,0.50],[0.50,0.50])	([0.18,0.24],[0.62,0.69])	([0.14,0.19],[0.70,0.76])
	$d_{2}$	([0.24,0.38],[0.50,0.62])	([0.60,0.60],[0.40,0.40])	([0.30,0.38],[0.50,0.62])
	$d_{3}$	([0.24,0.50],[0.50,0.50])	([0.38,0.47],[0.40,0.53])	([0.24,0.38],[0.50,0.62])
	$d_{4}$	([0.14,0.30],[0.50,0.62])	([0.38,0.47],[0.40,0.53])	([0.30,0.38],[0.50,0.62])
	$d_{5}$	([0.24,0.50],[0.50,0.50])	([0.13,0.18],[0.40,0.53])	([0.24,0.38],[0.50,0.62])

According to Step 4, use Eq. (6) to calculate the degree of proximity (${GN}_{i}$) between the weighted evaluation matrix (${\tilde{B}}_{i}$) and the positive ideal evaluation matrix (${\tilde{B}}^{+}$) of each alternative, and the calculation results are shown in Table 6. According to the results of the calculation in Step 4, the group utility value (${GNC}_{i}$) of each alternative can be calculated by using the Eq. (12) in Step 5, and the ranking based on the value of the group utility is shown in Table 8.

**Table 6:** Individual regret matrix for each alternative.

Evaluation matrix	Experts	$g_{1}$	$g_{2}$	$g_{3}$
${\tilde{R}}_{1}$	$d_{1}$	([0.19,0.25],[0.69,0.75])	([0.08,0.13],[0.75,0.82])	([0.05,0.14],[0.74,0.85])
	$d_{2}$	([0.05,0.09],[0.82,0.91])	([0.10,0.12],[0.81,0.85])	([0.05,0.09],[0.83,0.88])
	$d_{3}$	([0.12,0.25],[0.62,0.75])	([0.15,0.25],[0.63,0.75])	([0.03,0.06],[0.86,0.91])
	$d_{4}$	([0.02,0.04],[0.94,0.95])	([0.12,0.25],[0.63,0.75])	([0.02,0.04],[0.87,0.96])
	$d_{5}$	([0.07,0.09],[0.71,0.88])	([0.02,0.04],[0.88,0.93])	([0.12,0.19],[0.62,0.76])
${\tilde{R}}_{2}$	$d_{1}$	([0.09,0.19],[0.69,0.81])	([0.11,0.17],[0.69,0.77])	([0.05,0.11],[0.79,0.85])
	$d_{2}$	([0.07,0.15],[0.77,0.85])	([0.10,0.12],[0.85,0.88])	([0.15,0.24],[0.65,0.76])
	$d_{3}$	([0.05,0.07],[0.82,0.93])	([0.02,0.04],[0.89,0.96])	([0.12,0.24],[0.62,0.76])
	$d_{4}$	([0.07,0.19],[0.57,0.73])	([0.15,0.25],[0.63,0.75])	([0.09,0.19],[0.65,0.76])
	$d_{5}$	([0.12,0.25],[0.62,0.75])	([0.03,0.10],[0.73,0.80])	([0.07,0.12],[0.77,0.85])
${\tilde{R}}_{3}$	$d_{1}$	([0.12,0.25],[0.69,0.75])	([0.05,0.13],[0.75,0.82])	([0.10,0.14],[0.74,0.80])
	$d_{2}$	([0.02,0.04],[0.92,0.96])	([0.24,0.24],[0.76,0.76])	([0.09,0.19],[0.65,0.81])
	$d_{3}$	([0.12,0.15],[0.71,0.85])	([0.12,0.25],[0.71,0.75])	([0.02,0.04],[0.86,0.91])
	$d_{4}$	([0.07,0.12],[0.79,0.87])	([0.10,0.16],[0.63,0.75])	([0.15,0.24],[0.65,0.76])
	$d_{5}$	([0.07,0.09],[0.77,0.88])	([0.05,0.10],[0.48,0.61])	([0.09,0.24],[0.62,0.76])
${\tilde{R}}_{4}$	$d_{1}$	([0.15,0.25],[0.69,0.75])	([0.03,0.06],[0.85,0.90])	([0.07,0.11],[0.84,0.88])
	$d_{2}$	([0.12,0.24],[0.62,0.76])	([0.12,0.19],[0.81,0.81])	([0.02,0.09],[0.87,0.91])
	$d_{3}$	([0.09,0.19],[0.62,0.81])	([0.15,0.25],[0.63,0.75])	([0.09,0.19],[0.62,0.81])
	$d_{4}$	([0.07,0.15],[0.74,0.83])	([0.07,0.13],[0.81,0.87])	([0.15,0.24],[0.65,0.76])
	$d_{5}$	([0.05,0.07],[0.62,0.81])	([0.02,0.07],[0.79,0.89])	([0.09,0.24],[0.62,0.76])

**Table 7:** Individual positive ideal regret matrix.

Evaluation matrix	Experts	$g_{1}$	$g_{2}$	$g_{3}$
${\tilde{R}}^{+}$	$d_{1}$	([0.09,0.19],[0.69,0.81])	([0.03,0.06],[0.85,0.90])	([0.05,0.11],[0.84,0.88])
	$d_{2}$	([0.02,0.04],[0.92,0.96])	([0.10,0.12],[0.85,0.88])	([0.02,0.09],[0.87,0.91])
	$d_{3}$	([0.05,0.07],[0.82,0.93])	([0.02,0.04],[0.89,0.96])	([0.02,0.04],[0.86,0.91])
	$d_{4}$	([0.02,0.04],[0.94,0.95])	([0.07,0.13],[0.81,0.87])	([0.02,0.04],[0.87,0.96])
	$d_{5}$	([0.05,0.07],[0.77,0.88])	([0.02,0.04],[0.88,0.93])	([0.07,0.12],[0.77,0.85])

**Table 8:** Ranking of alternatives for the methodology in this paper.

Alternatives	${GN}_{i}$	Ranking	${GNC}_{i}$	Ranking	${GR}_{i}$	Ranking	${GRC}_{i}$	Ranking	$Q_{i}$	Ranking
$A_{1}$	0.7373	4	0.0000	4	0.8556	4	0.0000	4	0.0000	4
$A_{2}$	0.8357	2	0.9876	2	0.8756	3	0.4467	3	0.7171	2
$A_{3}$	0.8369	1	1.0000	1	0.9005	1	1.0000	1	1.0000	1
$A_{4}$	0.7822	3	0.4505	3	0.8874	2	0.7096	2	0.5801	3

Based on the group utility positive ideal evaluation matrix (${\tilde{B}}^{+}$) in Table 5, the individual regret matrix (${\tilde{R}}_{i}$) of each alternative is calculated by Step 6, and the results are shown in Table 6. The individual positive ideal regret matrix (${\tilde{R}}^{+}$) is determined by Step 7, and the results are shown in Table 7.

Based on Table 6 and Table 7, the degree of proximity (${GR}_{i}$) between the individual regret matrix (${\tilde{R}}_{i}$) and the positive ideal regret matrix (${\tilde{R}}^{+}$) of each alternative is calculated respectively according to Step 8. According to the results calculated in Step 8, the individual regret value (${GRC}_{i}$) based on the individual regret matrix can be calculated by using the formula (15) in Step 9, and the ranking based on ${GRC}_{i}$ are shown in Table 8.

Based on Step 10,α=0.5 is taken, and the value of the comprehensive measure ($Q_{i}$) of each alternative is calculated using Eq. (16), and the results of the calculation as well as the ranking based on $Q_{i}$ are shown in Table 8.

The patent quality of the alternatives is ranked according to Step 11. According to the calculation results in Table 8, it can be seen that the patent quality of the four alternative suppliers is ranked according to the value of the comprehensive measurement index as follows: ${A_{3}>A_{2}>A}_{4}>A_{1}$, so the patent quality of the four alternative suppliers is optimized according to the value of the comprehensive measurement index as $A_{3}$, followed by $A_{2}$.

Determine the compromise solution based on Step 12. According to Table 8, it can be seen that $Q\left(A_{3}\right)-Q\left(A_{2}\right)=0.2829<\frac{1}{3}$, $Q\left(A_{3}\right)-Q\left(A_{4}\right)=0.4191>\frac{1}{3}$,$Q\left(A_{4}\right)-Q\left(A_{1}\right)=0.4191>\frac{1}{3}$,$A_{3}$, and $A_{2}$ are both compromises and at this point, the ranking is ${\left\{A_{3},A_{2}\right\}>A}_{4}>A_{1}$.

Consider that the group utility value (${GNC}_{i}$) and the individual regret value (${GRC}_{i}$) are also measures, as can be seen in Table 8:

(1) The ranking result based on the group utility value (${GNC}_{i}$) is ${A_{3}>A_{2}>A}_{4}>A_{1}$, which is consistent with the ranking result based on the comprehensive measure;
(2) The ranking result based on the individual regret value (${GRC}_{i}$) is ${A_{3}>A_{4}>A}_{2}>A_{1}$, which is slightly different from the ranking result based on the comprehensive measure value, where the optimum is $A_{3}$ in both cases;
(3) When the decision maker makes decisions based on the group utility value (${GNC}_{i}$), $A_{2}$ is better than $A_{4}$, and when the decision maker makes decisions based on the individual regret value (${GRC}_{i}$), $A_{4}$ is better than $A_{2}$.

In summary, it can be concluded that the GDM method of normalized projection measure proposed in this paper is based on the group utility value (${GNC}_{i}$) the individual regret value (${GRC}_{i}$), and the comprehensive measure value ($Q_{i}$), the optimal is $A_{3}$.

### 6.2 Comparative analysis

#### (a) Comparison with existing projection measures and traditional VIKOR Methods.

To demonstrate the effectiveness and benefits of the GDM method presented in this paper, we will compare it with the GDM method that uses classical projection measures, normalized projection measures found in the literature [41], and Euclidean distance measures. The superiority of the new method is best seen through these comparisons. To begin with, we will substitute Eqs. (10) and (14) with the classical projection measure:


$${{GN}_{i}=Proj}_{{\tilde{B}}^{+}}\left({\tilde{B}}_{i}\right),i\in M$$ (17)

$${{GR}_{i}=Proj}_{{\tilde{R}}^{+}}\left({\tilde{R}}_{i}\right),i\in M$$ (18)

where ${Proj}_{{\tilde{B}}^{+}}\left({\tilde{B}}_{i}\right)=\frac{{\tilde{B}}_{i}{\tilde{B}}^{+}}{\left|{\tilde{B}}^{+}\right|}$, ${Proj}_{{\tilde{R}}^{+}}\left({\tilde{R}}_{i}\right)=\frac{{\tilde{R}}_{i}{\tilde{R}}^{+}}{\left|{\tilde{R}}^{+}\right|}$, and others are the same as Eqs. (10) and (14).

Based on Eqs. (17) and (18), the proximity (${GN}_{i}$) of the weighted evaluation matrix (${\tilde{B}}_{i}$) to the positive ideal evaluation matrix (${\tilde{B}}^{+}$) for each alternative of the GDM method based on the classical projection measure, the group utility value (${GNC}_{i}$), the proximity value (${GR}_{i}$) of the individual regret matrix (${\tilde{R}}_{i}$) to the individual positive ideal regret matrix (${\tilde{R}}^{+}$) for each alternative, the individual regret value (${GRC}_{i}$), and the comprehensive measure ($Q_{i}$) were calculated according to Steps 4 to 10, and the results of the calculations and the ordering are shown in Table 9.

**Table 9:** Ranking of alternatives for the classical projection method.

Alternatives	${GN}_{i}$	Ranking	${GNC}_{i}$	Ranking	${GR}_{i}$	Ranking	${GRC}_{i}$	Ranking	$Q_{i}$	Ranking
$A_{1}$	3.8756	2	0.8575	2	3.8970	4	0.0000	4	0.4288	3
$A_{2}$	3.7356	4	0.0000	4	3.9286	3	0.3948	3	0.1974	4
$A_{3}$	3.8394	3	0.6358	3	3.9771	1	1.0000	1	0.8179	2
$A_{4}$	3.8989	1	1.0000	1	3.9533	2	0.7031	2	0.8516	1

Similarly, replacing Eq. (10) with the normalized projection measure of literature [41] results in:


$${{GN}_{i}=NProj}_{{\tilde{B}}^{+}}\left({\tilde{B}}_{i}\right),i\in M$$ (19)

$${{GR}_{i}=NProj}_{{\tilde{R}}^{+}}\left({\tilde{R}}_{i}\right),i\in M$$ (20)

where ${NProj}_{{\tilde{B}}^{+}}\left({\tilde{B}}_{i}\right)=\frac{{\tilde{B}}_{i}{\tilde{B}}^{+}}{\left({\tilde{B}}_{i}{\tilde{B}}^{+}+\left|{\left|{\tilde{B}}_{i}\right|}^{2}-{\left|{\tilde{B}}^{+}\right|}^{2}\right|\right)}$, ${NProj}_{{\tilde{R}}^{+}}\left({\tilde{R}}_{i}\right)=\frac{{\tilde{R}}_{i}{\tilde{R}}^{+}}{\left({\tilde{R}}_{i}{\tilde{R}}^{+}+\left|{\left|{\tilde{R}}_{i}\right|}^{2}-{\left|{\tilde{R}}^{+}\right|}^{2}\right|\right)}$.

Based on Eqs. (19) and (20), the proximity (${GN}_{i}$) of the weighted evaluation matrix (${\tilde{B}}_{i}$) to the positive ideal evaluation matrix (${\tilde{B}}^{+}$) for each alternative of the GDM method based on the normalized projection measure of literature [41], the group utility value (${GNC}_{i}$), the proximity value (${GR}_{i}$) of the individual regret matrix (${\tilde{R}}_{i}$) to the individual positive ideal regret matrix (${\tilde{R}}^{+}$) for each alternative, the individual regret value (${GRC}_{i}$), and the comprehensive measure ($Q_{i}$) were calculated according to Steps 4–10, and the results of the calculations and the ordering are shown in Table 10.

**Table 10:** Ranking of alternatives for the normalized projection measure of literature [41].

Alternatives	${GN}_{i}$	Ranking	${GNC}_{i}$	Ranking	${GR}_{i}$	Ranking	${GRC}_{i}$	Ranking	$Q_{i}$	Ranking
$A_{1}$	0.8052	4	0.0000	4	0.9100	4	0.0000	4	0.0000	4
$A_{2}$	0.8901	1	1.0000	1	0.9271	3	0.6178	3	0.8089	2
$A_{3}$	0.8840	2	0.9273	2	0.9377	1	1.0000	1	0.9636	1
$A_{4}$	0.8414	3	0.4270	3	0.9308	2	0.7509	2	0.5889	3

Further replacing Eqs. (10) and (14) with the traditional Euclidean distance measure results in:


$${GN}_{i}=D\left({\tilde{B}}_{i},{\tilde{B}}^{+}\right)$$ (21)

$${GR}_{i}=D\left({\tilde{R}}_{i},{\tilde{R}}^{+}\right)$$ (22)

where $D\left({\tilde{B}}_{i},{\tilde{B}}^{+}\right)=\sum_{k=1}^{t}\sum_{j=1}^{n}\sqrt{\frac{1}{4}\left[{\left(\tau_{kj}^{il}{-\tau }_{kj}^{+l}\right)}^{2}+{\left(\tau_{kj}^{iu}{-\tau }_{kj}^{+u}\right)}^{2}+{\left(\upsilon_{kj}^{il}{-\upsilon }_{kj}^{+l}\right)}^{2}+{\left(\upsilon_{kj}^{iu}{-\upsilon }_{kj}^{+u}\right)}^{2}+{\left(\pi_{kj}^{il}{-\pi }_{kj}^{+l}\right)}^{2}+{\left(\pi_{kj}^{iu}{-\pi }_{kj}^{+u}\right)}^{2}\right]}$, $D\left({\tilde{R}}_{i},{\tilde{R}}^{+}\right)=\sum_{k=1}^{t}\sum_{j=1}^{n}\sqrt{\frac{1}{4}\left[{\left(\varphi_{kj}^{il}{-\varphi }_{kj}^{+l}\right)}^{2}+{\left(\varphi_{kj}^{iu}{-\varphi }_{kj}^{+u}\right)}^{2}+{\left(\phi_{kj}^{il}{-\phi }_{kj}^{+l}\right)}^{2}+{\left(\phi_{kj}^{iu}{-\phi }_{kj}^{+u}\right)}^{2}+{\left(\varepsilon_{kj}^{il}{-\varepsilon }_{kj}^{+l}\right)}^{2}+{\left(\varepsilon_{kj}^{iu}{-\varepsilon }_{kj}^{+u}\right)}^{2}\right]}$,$\pi_{kj}^{il}=1-\tau_{kj}^{iu}-\upsilon_{kj}^{iu}$,$\pi_{kj}^{iu}=1-\tau_{kj}^{il}-\upsilon_{kj}^{il}$,$\pi_{kj}^{+l}=1-\tau_{kj}^{+u}-\upsilon_{kj}^{+u}$,$\pi_{kj}^{+u}=1-\tau_{kj}^{+l}-\upsilon_{kj}^{+l}$,$\varepsilon_{kj}^{il}=1-\varphi_{kj}^{iu}-\phi_{kj}^{iu}$,$\varepsilon_{kj}^{iu}=1-\varphi_{kj}^{il}-\phi_{kj}^{il}$,$\varepsilon_{kj}^{+l}=1-\varphi_{kj}^{+u}-\phi_{kj}^{+u}$,$\varepsilon_{kj}^{+u}=1-\varphi_{kj}^{+l}-\phi_{kj}^{+l}$.

Let ${GN}^{+}=\max_{1\leq i\leq m}\left\{{GN}_{i}\right\}$, ${GN}^{-}=\min_{1\leq i\leq m}\left\{{GN}_{i}\right\}$, ${GR}^{+}=\max_{1\leq i\leq m}\left\{{GR}_{i}\right\}$, ${GR}^{-}=\min_{1\leq i\leq m}\left\{{GR}_{i}\right\}$.

Based on Eqs. (21) and (22), we know that the smaller ${GN}_{i}$ and the larger ${GR}_{i}$ indicates that the alternative $A_{i}$ is better. Therefore we replaceEqs. (11) and (15):


$${GNC}_{i}=\left\{ \begin{array}{c} \frac{{GN}_{i}-{GN}^{-}}{{{GN}^{+}-GN}^{-}},if{GN}^{+}\neq {GN}^{-}\\ 1,if{GN}^{+}={GN}^{-} \end{array}\right.,i\in M$$ (23)

$${GRC}_{i}=\left\{ \begin{array}{c} \frac{{GR}_{i}-{GR}^{-}}{{{GR}^{+}-GR}^{-}},if{GR}^{+}\neq {GR}^{-}\\ 1,if{GR}^{+}={GR}^{-} \end{array}\right.,i\in M$$ (24)

Based on the GDM method of Eqs. (21) to (24) Euclidean distance measure, the Euclidean distance value (${GN}_{i}$) of the weighted evaluation matrix (${\tilde{B}}_{i}$) and positive ideal evaluation matrix _(_${\tilde{B}}^{+}$_)_ for each alternative, the group utility value (${GNC}_{i}$), the Euclidean distance value (${GR}_{i}$) of the individual regret matrix (${\tilde{R}}_{i}$) and the individual positive ideal regret matrix ${\tilde{R}}^{+}$ for each alternative, the individual regret value (${GRC}_{i}$), and the value of the comprehensive measure ($Q_{i}$) was calculated based on Steps 4–10, and the results of the calculations and the ordering are shown in Table 11.

**Table 11:** Ranking of alternatives for the Euclidean distance measure.

Alternatives	${GN}_{i}$	Ranking	${GNC}_{i}$	Ranking	${GR}_{i}$	Ranking	${GRC}_{i}$	Ranking	$Q_{i}$	Ranking
$A_{1}$	4.9145	4	0.0000	4	1.7120	4	0.0000	4	0.0000	4
$A_{2}$	3.7193	2	0.7765	2	1.5508	3	0.3888	3	0.5826	2
$A_{3}$	3.3753	1	1.0000	1	1.2973	1	1.0000	1	1.0000	1
$A_{4}$	4.1395	3	0.5035	3	1.5315	2	0.4352	2	0.4693	3

From Table 9 above:

(1) Based on the comprehensive measure ($Q_{i}$), the ranking result based on the classical projection method is ${A_{4}>A_{3}>A}_{1}>A_{2}$, and the optimum is $A_{4}$, which is inconsistent with the projection method in this paper. The ranking result based on the compromise scheme is ${\left\{A_{4},A_{3}\right\}>A}_{1}>A_{2}$, which is also inconsistent with the ranking result of the projection method in this paper.
(2) Based on the group utility value (${GNC}_{i}$), the ranking result based on the classical projection method is ${A_{4}>A_{1}>A}_{3}>A_{2}$, and the optimum is $A_{4}$, which is inconsistent with the ranking result of the projection method in this paper. Based on the individual regret value (${GRC}_{i}$) the ranking result of the optimal program is $A_{3}$, which is consistent with the projection method of this paper, but the overall ranking result is ${A_{3}>A_{4}>A}_{2}>A_{1}$, and the ranking result of the other alternatives except $A_{3}$ is inconsistent with the ranking result of the projection method of this paper.

From Table 10 above:

(1) Based on the integrated measure value ($Q_{i}$), the ranking result of the projection method based on the literature [41] is ${A_{3}>A_{2}>A}_{4}>A_{1}$, optimal is $A_{3}$, and the ranking result based on the compromise scheme is ${\left\{A_{3},A_{2}\right\}>A}_{4}>A_{1}$, which is consistent with the ranking result of the projection method in this paper, and it is reflected from the side that the normalized projection measure method based on the literature [41] is supported by this paper’s method based on the integrated measure ($Q_{i}$).
(2) Based on the group utility value (${GNC}_{i}$), based on the literature [41] projection measurement method ranking result is ${A_{2}>A_{3}>A}_{4}>A_{1}$, which is inconsistent with the ranking result of this paper’s projection method. Based on the individual regret value $GRC_{i}$, the ranking result based on the literature [41] projection method is ${A_{3}>A_{4}>A}_{2}>A_{1}$, which is inconsistent with the ranking result of this paper.

From Table 11 above:

(1) According to the comprehensive measure ($Q_{i}$), the ranking result based on the Euclidean distance measure method is ${A_{4}>A_{3}>A}_{1}>A_{2}$, the optimal is $A_{4}$, which is consistent with the ranking result of the projection method in this paper, and it reflects that the ranking result based on the Euclidean distance measure method based on the comprehensive measure ($Q_{i}$) is supportive of the projection method in this paper, and the ranking result based on the compromise scheme is ${\left\{A_{4},A_{3}\right\}>A}_{1}>A_{2}$, which is inconsistent with the ranking result of the projection method in this paper.
(2) According to the group utility value (${GNC}_{i}$), the ranking result based on the Euclidean distance measurement method is ${A_{4}>A_{1}>A}_{3}>A_{2}$, which is consistent with the ranking result of the projection method in this paper, and it reflects that the method based on the Euclidean distance measurement method is supportive of the projection method in this paper from the side. Based on the individual regret value (${GRC}_{i}$), the ranking result based on the Euclidean distance measurement method is ${A_{3}>A_{4}>A}_{2}>A_{1}$, which is inconsistent with the ranking result of the projection method in this paper.

In summary, it can be seen that the ranking results of this paper’s normalized projection measure based on the comprehensive measure value ($Q_{i}$) are consistent with the ranking results of the normalized projection measure and Euclidean distance measure in the literature [41], and the ranking results are inconsistent with the ranking results of the classical projection measure, which illustrates the validity and feasibility of this paper’s normalized projection measure, and exposes the defects of the classical projection measure. In the ranking based on group utility value (${GNC}_{i}$), individual regret value (${GRC}_{i}$), and comprehensive measure ($Q_{i}$), the optimal results of the ranking of normalized projection measure and Euclidean distance measure in this paper is $A_{3}$, while the optimal results of the ranking based on group utility value (${GNC}_{i}$) and comprehensive measure ($Q_{i}$) in the normalized projection measure based on the literature [41] is $A_{3}$ and the optimal results of the ranking based on group regret value (${GRC}_{i}$) is $A_{2}$. This highlights the advantages of the normalized projection measurement method in this paper.

**(b) Comparison with Other Classical Methods**

To further illustrate the reliability of the results in this paper, this study will further conduct a comparative analysis with the existing classical Weighted Aggregated Sum Product Assessment (WASPAS) and TOPSIS methods on the evaluation results of the above example. The basic steps of the WASPAS method and the TOPSIS method based on IVIF are as follows:

Basic Steps of the WASPAS method based on IVIF:

**Step 1:** Determine the decision problem. This includes identifying alternative solutions, evaluation attributes, and the group of decision-makers. For detailed information, refer to the above example.

**Step 2:** Establish the IVIF number evaluation matrix ${\tilde{A}}_{i}={\left({\tilde{a}}_{kj}^{i}\right)}_{t\times n}$ by collecting the evaluation information of decision-makers. The evaluation information of decision-makers is expressed in IVIF numbers.

**Step 3:** Determine the weights w of the evaluation attributes

**Step 4:** Calculate the relative importance ${WSM}_{i}$ based on the weighted sum model (WSM) and the relative importance ${WPM}_{i}$ based on the weighted product model (WPM), with the calculation formulas as follows:


$${WSM}_{i}=\sum_{k=1}^{t}\sum_{j=1}^{n}{\tilde{a}}_{kj}^{i}\cdot w_{j}$$

$${WPM}_{i}=\sum_{k=1}^{t}\prod_{j=1}^{n}{\left({\tilde{a}}_{kj}^{i}\right)}^{w_{j}}$$

**Step 5:** Calculate the total relative importance of WASPAS. Based on the calculation results of the Weighted Sum Model (WSM) and the Weighted Product Model (WPM), use the generalized WASPAS criterion formula to calculate the total relative importance ${WASPAS}_{i}$ of each alternative option, and the calculation formula is as follows:


$${WASPAS}_{i}=\beta \cdot {WSM}_{i}+(1-\beta )\cdot {WPM}_{i}=\left(\left[\vartheta_{i}^{l},\vartheta_{i}^{u}\right],\left[\rho_{i}^{l},\rho_{i}^{u}\right]\right)$$

where β is a parameter, and the value of β determines the degree of emphasis of the WASPAS method on the Weighted Sum Model (WSM) and the Weighted Product Model (WPM). Here, the value of β is taken as β=0.5.

**Step 6:** Calculate the score level ($S_{i}$) of the total relative importance ${WASPAS}_{i}$ of each alternative option, and the calculation formula is as follows:


$$S_{i}=\frac{\vartheta_{i}^{l}-\rho_{i}^{l}+\vartheta_{i}^{u}-\rho_{i}^{u}}{2},\forall S_{i}\in \left[-1,1\right]$$

**Step 7:** Ranking of alternatives. Ranking of alternatives according to the score level ($S_{i}$). A larger score level ($S_{i}$) indicates a better alternative ($A_{i}$).

Basic Steps of the TOPSIS method based on IVIF

**Step 1:** Determine the decision problem. This includes identifying alternative solutions, evaluation attributes, and the group of decision-makers. For detailed information, refer to the above example.

**Step 2:** Establish the IVIF number evaluation matrix ${\tilde{A}}_{i}={\left({\tilde{a}}_{kj}^{i}\right)}_{t\times n}$ by collecting the evaluation information of decision-makers. The evaluation information of decision-makers is expressed in IVIF numbers.

**Step 3:** Determine the weights w of the evaluation attributes and calculate the weighted evaluation matrix ${\tilde{B}}_{i}={\left({\tilde{b}}_{kj}^{i}\right)}_{t\times n}$ of the evaluation attributes.

**Step 4:** According to the idea of the classical TOPSIS method, determine the positive and negative ideal solutions:


$${\tilde{B}}^{+}={\left({\tilde{b}}_{kj}^{+}\right)}_{t\times n}=\left(\left[\tau_{kj}^{+l},\tau_{kj}^{+u}\right],\left[\upsilon_{kj}^{+l},\upsilon_{kj}^{+u}\right]\right)=\left(\left[\max_{i}\tau_{kj}^{il},\max_{i}\tau_{kj}^{iu}\right],\left[\left[\min_{i}\upsilon_{kj}^{il},\min_{i}\upsilon_{kj}^{iu}\right]\right]\right)$$

$${\tilde{B}}^{-}={\left({\tilde{b}}_{kj}^{-}\right)}_{t\times n}=\left(\left[\tau_{kj}^{-l},\tau_{kj}^{-u}\right],\left[\upsilon_{kj}^{-l},\upsilon_{kj}^{-u}\right]\right)=\left(\left[\min_{i}\tau_{kj}^{il},\min_{i}\tau_{kj}^{iu}\right],\left[\left[\max_{i}\upsilon_{kj}^{il},\max_{i}\upsilon_{kj}^{iu}\right]\right]\right)$$

**Step 5:** According to the idea of the classical TOPSIS method, use the following formula to calculate the distances between the evaluation matrices of each alternative and the positive and negative ideal solutions:


$$D_{i}^{+}=\sum_{k=1}^{t}\sum_{j=1}^{n}\sqrt{\left[{\left(\tau_{kj}^{il}{-\tau }_{kj}^{+l}\right)}^{2}+{\left(\tau_{kj}^{iu}{-\tau }_{kj}^{+u}\right)}^{2}+{\left(\upsilon_{kj}^{il}{-\upsilon }_{kj}^{+l}\right)}^{2}+{\left(\upsilon_{kj}^{iu}{-\upsilon }_{kj}^{+u}\right)}^{2}+{\left(\pi_{kj}^{il}{-\pi }_{kj}^{+l}\right)}^{2}+{\left(\pi_{kj}^{iu}{-\pi }_{kj}^{+u}\right)}^{2}\right]}$$

$$D_{i}^{-}=\sum_{k=1}^{t}\sum_{j=1}^{n}\sqrt{\left[{\left(\tau_{kj}^{il}{-\tau }_{kj}^{-l}\right)}^{2}+{\left(\tau_{kj}^{iu}{-\tau }_{kj}^{-u}\right)}^{2}+{\left(\upsilon_{kj}^{il}{-\upsilon }_{kj}^{-l}\right)}^{2}+{\left(\upsilon_{kj}^{iu}{-\upsilon }_{kj}^{-u}\right)}^{2}+{\left(\pi_{kj}^{il}{-\pi }_{kj}^{-l}\right)}^{2}+{\left(\pi_{kj}^{iu}{-\pi }_{kj}^{-u}\right)}^{2}\right]}$$

**Step 6:** Calculate the closeness coefficient:


$$U_{i}=\frac{D_{i}^{-}}{D_{i}^{+}+D_{i}^{-}}$$

**Step 7:** Ranking of alternatives. Ranking of alternatives according to the closeness coefficient ($U_{i}$). A larger closeness coefficient ($U_{i}$) indicates a better alternative ($A_{i}$).

For the specific implementation details of the WASPAS method based on IVIF, please refer to the literature [47]. For the specific implementation details of the TOPSIS method based on IVIF, you can refer to the existing literature [48]. The calculation results of the measurement indicators and the rankings of each method are shown in Table 12.

**Table 12:** Comparison with other classical methods.

Alternatives	WASPAS method	Ranking	TOPSIS method	Ranking	Method of this paper	Ranking
Score degree ($S_{i}$)	Closeness coefficient ($U_{i}$)	Comprehensive measure ($Q_{i}$)
$A_{1}$	0.0382	4	0.4189	4	0.0000	4
$A_{2}$	0.1028	2	0.5439	2	0.7171	2
$A_{3}$	0.1267	1	0.6068	1	1.0000	1
$A_{4}$	0.0901	3	0.5271	3	0.5801	3

As can be seen from Table 12, the rankings of the alternatives in the decision-making results of the method in this study are completely consistent with those of the classical WASPAS and TOPSIS methods. This indicates the consistency between the decision-making results of the example using the method in this study and those of the existing mature methods, thereby once again confirming the reliability and credibility of the ranking results of the decision-making method proposed by us.

### 6.3 Experimental analysis of the decision-making mechanism coefficient

**Fig 2:**
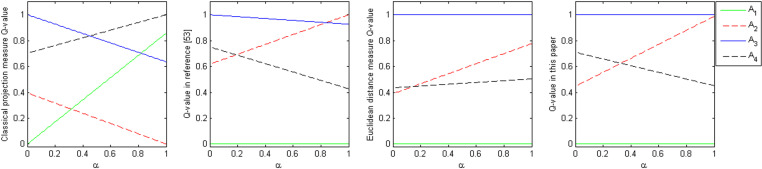
Dynamics and ranking of the comprehensive measures of alternatives with the decision-making mechanism coefficient.

To more intuitively analyze the dynamic change of the comprehensive measure ($Q_{i}$) with the decision-making mechanism coefficient (α) for the four alternatives in this paper’s method, the curves of the dynamic change of the comprehensive measure ($Q_{i}$) with the decision-making mechanism coefficient (α) based on the classical projection measure, the normalized projection measure of the literature [41], the Euclidean distance measure, and this paper’s measure were made respectively in Fig 2.

From Fig 2 can be seen:

(1) This paper normalized projection measurement method and based on the Euclidean distance measurement method with the change of the decision-making mechanism coefficient (α), the alternatives based on the comprehensive measurement value ($Q_{i}$) ranking have roughly the same rule of change, the optimal alternative is always $A_{3}$, the worst alternative is $A_{1}$, the ranking relationship has a certain degree of stability, but also from the side of the euclidean distance measurement method based on the method of this paper is to support the method.
(2) Based on the classical projection measurement method, with the change of the decision-making mechanism coefficient (α), the ranking of the alternatives based on the comprehensive measurement ($Q_{i}$) is unstable, which exposes the disadvantage of the classical projection measurement method.
(3) Based on the literature [41] normalized projection measurement method with the change of the decision-making mechanism coefficient (α), the alternatives based on the comprehensive measurement ($Q_{i}$) ranking change as follows: when α<0.85, the optimal program is $A_{3}$, when α≥0.85, the optimal program is $A_{2}$.

A comprehensive analysis of the above can conclude that the method of this paper has good stability, and by comparing with the classical projection measurement method, the literature [41] normalized projection measurement method, and the Euclidean distance measurement method, it shows that the feasibility and effectiveness of the GDM method of normalized projection measurement proposed in this paper.

### 6.4 Comparative analysis of dynamic experiments

Since the previous experimental analysis was conducted using the same data example, it may not be sufficient to support the argument. Therefore, to further illustrate the advantages of the model constructed in this paper, two dynamic experiments will be conducted to supplement the previous results effectively.

#### (1) Dynamic Experiment I.

**Fig 3:**
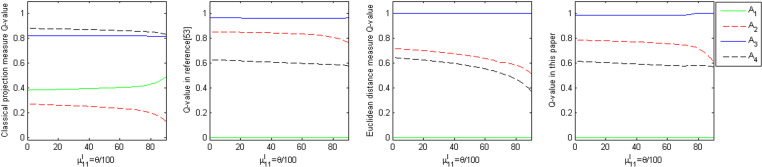
Dynamics and ranking of the comprehensive measure of alternative with parameter θ.

Known evaluation matrix ${\tilde{A}}_{1}$ in the element ${\tilde{a}}_{11}=([0.8,0.9],[0.1,0.1])$, where $\mu_{11}^{l}=0.8$, so that $\mu_{11}^{l}=\frac{\theta }{100}$, θ∈[0,90], keep the value of other elements in ${\tilde{A}}_{1}$ unchanged, so that θ from 0 constantly increasing to 90, plotting the alternatives of the comprehensive measure $Q_{i}$ with the parameters of the dynamic change of θ, the dynamic change of the curve reflects the relationship between the advantages and disadvantages of the alternatives, and its dynamic changes as shown in Fig 3.

From Fig 3 can be seen:

(1) The comprehensive measures ($Q_{i}$) of alternatives are very close to each other based on the classical projection measure as the parameter θ changes, and the comprehensive measures ($Q_{i}$) of alternatives $A_{2}$ and $A_{4}$ are very close to each other based on the Euclidean distance measure as the parameter θ changes.
(2) The normalized projection measure, as presented in the literature [41], and the method proposed in this paper, both measure the ranking distinction of each alternative based on an integrated measure value. However, the method proposed in this paper is more straightforward and highlights the advantages of using the normalized projection measure.

To sum up, according to the comprehensive measure ($Q_{i}$), the GDM approach based on the normalized projection measure in the literature [41] and Euclidean distance measure, and the ranking of the alternatives of the GDM method with the normalized projection measure measures in this paper, are consistent. The optimal alternatives identified are all $A_{3}$. In contrast, the optimal choice among GDM methods based on classical projection measures is $A_{4}$, highlighting the drawback of classical projection measure methods, but also reflecting the feasibility and effectiveness of this paper’s normalized projection measure from the side.

#### (2) Dynamic Experiment II.

**Fig 4:**
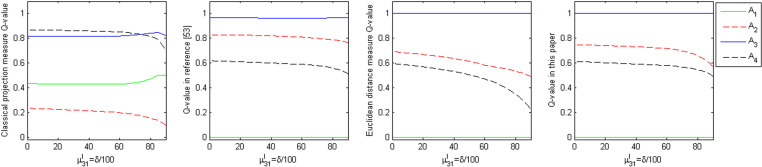
Dynamics and ranking of the comprehensive measure of alternative with parameter . δ.

Known evaluation matrix ${\tilde{A}}_{1}$ in the element ${\tilde{a}}_{11}=([0.6,0.9],[0.1,0.1])$, where $\mu_{31}^{l}=0.6$, so that $\mu_{31}^{l}=\frac{\delta }{100}$, δ∈[0,90], keep the value of other elements in ${\tilde{A}}_{1}$ unchanged, so that δ from 0 constantly increasing to 90, plotting the alternatives of the comprehensive measure ($Q_{i}$) with the parameters of the dynamic change of δ, the dynamic change of the curve reflects the relationship between the advantages and disadvantages of the alternatives, and its dynamic changes as shown in Fig 4.

From Fig 4 can be seen:

(1) the classical projection measure method with the change of parameter δ, the comprehensive measure ($Q_{i}$) of alternatives $A_{3}$ and $A_{4}$ is very close to each other and $Q\left(A_{4}\right)>Q\left(A_{3}\right)$ when δ<0.74, which is inconsistent with the literature [41] normalized projection measure, Euclidean distance measure, and the normalized projection measure method in this paper.
(2) The normalization projection measure, Euclidean distance measure, and the normalized projection measure method discussed in this paper are consistent with the ranking relationship of the alternatives, regardless of parameter δ changes. The ranking relationship remains stable, which indicates the reliability of the normalized projection measure and Euclidean distance measure methods mentioned in the literature [41] and supports the normalized projection measure method presented in this paper. This illustrates the feasibility and validity of the method of this paper.

## 7. Conclusion

This study has proposed an evaluation method for enterprise patent quality in the environment of IVIF information. The proposed method has five important contributions, which are detailed as follows:

(1) The subtraction operation rule for IVIF numbers has been defined. On the one hand, this contribution provides a specific method for calculating the regret matrix in this study. On the other hand, it further improves the theoretical basis of IVIF numbers.
(2) A new generalized standardized projection measure formula has been put forward. This formula can be directly used to measure the closeness degree between two IVIF number vectors or matrices, which overcomes the defects of classical projection and existing standardized projection measures and has a better comprehensive measurement ability.
(3) By utilizing the proposed generalized standardized projection measure formula, the VIKOR technology has been extended, and a new GDM method based on the evaluation information in the form of IVIF numbers has been established.
(4) Through the subtraction operation of IVIF numbers defined in this study, a specific group regret matrix for the VIKOR method has been provided. By simultaneously integrating the utility matrix and the regret matrix, the comprehensive ability of the VIKOR method has been enhanced, and the reliability of the model results has been improved.
(5) The new VIKOR-based GDM method established in this paper has been applied to an example of enterprise patent quality assessment. Through data experiments, the feasibility and effectiveness of the method in this paper have been demonstrated.

This method can handle preference ranking in decision science. This method is expected to be widely applied in other related fields, including service quality evaluation, hardware quality evaluation, software quality evaluation, and so on. Certainly, the method proposed in this paper also has some limitations. Firstly, in this study, only the evaluation information in the form of IVIF numbers has been considered, while other forms of evaluation information, including triangular fuzzy numbers, trapezoidal fuzzy numbers, linguistic fuzzy information, or neutrosophic cubic fuzzy sets, etc., have not been taken into account. Future research intends to extend IVIF information to other fuzzy information. Secondly, in this paper, the advantages of the proposed standardized projection measure have only been verified through one example. This is a limitation. Future work should discuss the advantages of enterprise patent quality assessment based on the standardized projection measure. Thirdly, the new VIKOR-based GDM method has only been applied to the assessment of enterprise patent quality. Future research intends to extend this application to other fields.
